# R-Ras Regulates Migration through an Interaction with Filamin A in Melanoma Cells

**DOI:** 10.1371/journal.pone.0011269

**Published:** 2010-06-23

**Authors:** Joanna E. Gawecka, Genevieve S. Griffiths, Barbro Ek-Rylander, Joe W. Ramos, Michelle L. Matter

**Affiliations:** 1 Natural Products and Cancer Biology, Cancer Research Center of Hawaii, University of Hawaii at Manoa, Honolulu, Hawaii, United States of America; 2 Department of Cell and Molecular Biology, John A. Burns School of Medicine, University of Hawaii at Manoa, Honolulu, Hawaii, United States of America; 3 Division of Pathology, Department of Laboratory Medicine, Karolinska Institutet, Karolinska University Hospital, Huddinge, Sweden; University of Birmingham, United Kingdom

## Abstract

**Background:**

Changes in cell adhesion and migration in the tumor microenvironment are key in the initiation and progression of metastasis. R-Ras is one of several small GTPases that regulate cell adhesion and migration on the extracellular matrix, however the mechanism has not been completely elucidated. Using a yeast two-hybrid approach we sought to identify novel R-Ras binding proteins that might mediate its effects on integrins.

**Methods and Findings:**

We identified Filamin A (FLNa) as a candidate interacting protein. FLNa is an actin-binding scaffold protein that also binds to integrin β1, β2 and β7 tails and is associated with diverse cell processes including cell migration. Indeed, M2 melanoma cells require FLNa for motility. We further show that R-Ras and FLNa interact in co-immunoprecipitations and pull-down assays. Deletion of FLNa repeat 3 (FLNaΔ3) abrogated this interaction. In M2 melanoma cells active R-Ras co-localized with FLNa but did not co-localize with FLNa lacking repeat 3. Thus, activated R-Ras binds repeat 3 of FLNa. The functional consequence of this interaction was that active R-Ras and FLNa coordinately increased cell migration. In contrast, co-expression of R-Ras and FLNaΔ3 had a significantly reduced effect on migration. While there was enhancement of integrin activation and fibronectin matrix assembly, cell adhesion was not altered. Finally, siRNA knockdown of endogenous R-Ras impaired FLNa-dependent fibronectin matrix assembly.

**Conclusions:**

These data support a model in which R-Ras functionally associates with FLNa and thereby regulates integrin-dependent migration. Thus in melanoma cells R-Ras and FLNa may cooperatively promote metastasis by enhancing cell migration.

## Introduction

Tumor cell metastasis causes 90% of cancer deaths [1]. The adhesive and migratory abilities of tumor cells and the tumor microenvironment are key components involved in cancer metastasis [2], [3]. Changes in cell-extracellular matrix (ECM) and cell-cell interactions are associated with the progression of primary melanomas to malignancy. Moreover, altered expression of integrins, ICAM, MelCam, cadherins and ECM can lead to increased melanoma invasiveness and metastatic spread [1]. Integrin receptors are heterodimers of α and β subunits that are integral in cell adhesion, cell migration, proliferation and programmed cell death. Integrin binding transmits signals into the cell that result in cytoskeletal re-organization, gene expression and cell differentiation. Moreover, signals from within the cell can regulate integrin ligand binding affinity (integrin activation) and cell adhesion. Currently at least 21 proteins are reported to bind to one or more of the integrin β tails and modulate cell behaviors including cell migration, adhesion, and fibronectin matrix assembly [4]. Furthermore, isolated integrin β tails can activate downstream signaling molecules and regulate actin cytoskeleton assembly [5]. As yet, the mechanism by which integrin β tails function in signal transduction to modulate changes in cell adhesive behavior has not been completely elucidated.

Filamin A (FLNa), a member of the non-muscle actin binding protein family, acts as a molecular scaffold protein that regulates signaling events involved in cell shape and cell migration by binding to β integrin tails, adaptor proteins and second messengers [6]. FLNa is required for melanoma cell migration [7] and melanoma cells that do not express FLNa do not migrate [7], [8]. In addition, FLNa is necessary for tumor cell metastasis and vascular remodeling [9], [10], [11], [12]. The cytoskeletal proteins talin and kindlin can bind integrin β tails and increase integrin activation [13]. Interestingly, talin and kindlin bind a site on the integrin tail corresponding to the sequence NPxY that overlaps with that of filamin [13]. Thus changes in integrin function in response to signaling pathways may be mediated in part by these integrin tail-binding proteins.

The oncogene R-Ras is a small GTPase that affects cell processes such as adhesion, migration, proliferation and survival [14], [15], [16]. R-Ras enhances integrin-mediated cell adhesion by increasing the affinity and avidity of integrins. Moreover, activated R-Ras promotes cell migration of breast and cervical epithelial cells [17], [18], [19], [20]. R-Ras induced cell migration promotes changes in the cytoskeleton through the activation of the GTPases Rho and Rac [21], [22], [23]. Thus, R-Ras is a regulator of cell adhesion and cell migration, however the mechanism by which it affects integrin function remains incompletely understood.

We used yeast 2 hybrid screens to identify novel binding partners of R-Ras that could mediate its effects on integrin function. We demonstrate an association between R-Ras and FLNa and that the resulting complex can significantly enhance melanoma migration and increase integrin activation and fibronectin matrix assembly. These data suggest that R-Ras promotes integrin-mediated melanoma cell migration and enhances fibronectin matrix assembly through a previously unknown association with FLNa.

## Materials and Methods

### Plasmid Constructs

Full-length human R-Ras was cloned into EcoRI and SmaI-sites of pBTM116 to yield BTM116-R-Ras. To construct an R-Ras-bait lacking the CAAX-box, the 3′-terminal of R-Ras-CAAX from pSKII was cleaved with XbaI, blunted with Klenow fragment and cleaved with SacII. This fragment was cloned into BTM116-R-Ras that had been cleaved with BamHI, blunted with Klenow fragment and cleaved with SacII. Lamin C cloned into pBTM116 was used as a negative control (BTM116-lamin).

### Library Screening

To screen for proteins that bind to R-Ras, the yeast two-hybrid system was used as previously described (29). Yeast strain L40 transformed with pBTM116-R-Ras-CAAX was grown in 5 ml UTL at 30°C overnight and diluted to 100 ml with UTL media and grown at 30°C overnight. On day 3 the yeast culture (100 ml) was added to 650 ml YPAD medium (20 g/L Difco peptone; 10 g/L yeast extract, 2% glucose, 40 µg/ml adenine) and grown to density of OD_600_ = 0.6. After centrifugation at 5,000 rpm for 5 min the pellet was washed with H_2_O, centrifuged, and suspended in 100 mM Lithium acetate (LiAc) pH 7.0, 0.5 x TE (1M EDTA, 0.001M Tris HCL, pH 7.5) and incubated for 10 min at room temp. Yeast suspension (20 ml), 1.5 ml sheared salmon sperm DNA (10 µg/ml; Oncor, Inc. Gaithersburg MD), 500 µg of the mouse embryo cDNA library in pVP16 and 140 ml of the 100 mM LiAc/TE, 40% PEG3350 (Sigma Chemical Co., St. Louis MO) was shaken for 30 min at 30°C. The mouse embryo cDNA library was a gift from S.M. Hollenberg (St. Luke's Medical Center Chicago, IL). The yeast suspension was then heat-shocked for 7 min at 42°C, washed with H_2_O, resuspended in 1 L YPD and shaken for 1 h at 30°C. Transformed yeast cells were resuspended in 20 ml H_2_O and plated. 10^6^ transformants were plated onto 20 THULL-15 cm plates. One µl and 10 µl aliquots were plated onto UTL-plates in order to calculate the transformation efficiency. After 6 days of growth at 30°C, 300 colonies were picked and streaked onto new THULL-plates. 200 his+clones were streaked on UTL-plates and incubated for 24 h at 30°C. β-gal activity of individual colonies was examined in a filter-lift assay as previously described [24].

### Yeast DNA Preparation

Yeast DNA was prepared by the Li-CL-method as previously described [25] from the positive 36 clones as examined by β-gal assay.

### Clones Sequenced

5 µl of yeast DNA was used in PCR-reaction with 30 pmol VP16 F-primer (GCT GAT ATG GCC GAC TTC) and 30 pmol M13 primer (GTA AAA CGA CGG CCA GT), 10 nmol dNTPs+1 µl Taq polymerase.

### Stable Cell Lines and Cell Transfection

Human melanoma cells (M2) were cultured as previously described (6). For stable cell lines pcDNA3 vector, pcDNA3-FLNa (FLNa) or pcDNA3-FLNaΔ3 plasmids were each transfected into FLNa-deficient M2 cells [7] by LipofectAMINE 2000 (Invitrogen, San Diego, CA) and G418 (500 µg ml^−1^) was added to media. β-Gal or R-Ras G38V, R-RAS T43N or His-tagged plasmids were transiently transfected using Lipofectamine 2000 manufacturer's protocol.

### Glutathione S-transferase (GST) protein construction and FLNa fragment pulldown assay

Human FLNa fragments were a gift from Dr. Jonathan Lee and fragments were purified as previously described (University of Ottawa; [26].

### Deletion of FLNa Repeat 3

His-FLNa was cleaved using PacI and BstBI. PCR was performed with FLNa deletion primer 1 and 2 (having PacI and BstBI restriction sites; primer 1: 5′CGC CC AAA CTG AA CC; primer 2: 3′CCT ACA CTG TCA CTG T). PCR was performed using 10 ng FLNa, taq polymerase, 5 pmol deletion primer 1 and 2 for 30 cycles. Cleaved FLNa and the deletion fragment obtained by PCR were run on a low melt agarose gel and purified by Wizard PCR DNA purification kit. Ligation of 440 ng cleaved FLNa + 50 ng deletion fragment was performed at 16°C overnight. FLNaΔ3 was cloned into pcDNA3.

### Integrin Activation and Flow Cytometry

Analytical two-color flow cytometry was carried out as previously described [27]. Briefly, 24 h post-transfection, M2 cells were harvested and analyzed for transfection efficiency (GFP) and integrin binding to 3FN-(9–11). In brief, for each transfection, harvested cells were divided into three tubes. The three preparations were used to assay for binding to 3FN-(9–11) alone, binding in the presence of EDTA (10 mM), or carried out in the presence of MnCl_2_. Integrin activation was quantified as an activation index (AI) as defined [27]. AI = 100*(F–F_0_)/(F_m_–F_0_), where **F** represents the geometric mean fluorescence (GMF) of 3Fn-(9–11) binding alone, **F_0_** is the GMF of 3FN-(9–11) binding in the presence of EDTA (10 mM), and **F_m_** is the GMF of 3Fn-(9–11) binding in the presence of MNCL_2_.

### Cell Migration Assays

Cell migration assays were carried out as previously described [17]. Briefly, lower sides of transwell filters (8.0 µm pore size; Costar, Cambridge MA) were coated overnight 4°C with 40 µg/ml human plasma FN. Cells (5×10^4^ cells/filter) were plated on the uncoated top side of the filters, incubated for 2 h (37°C) and uncoated top side of each filter was swabbed with a cotton-tip applicator to remove cells that had not migrated through [21]. The filters were fixed with 2% glutaraldehyde (30 min) and β-Galactosidase activity (β-gal) in only the transfected cells that had migrated through the filters, was determined by staining the bottom side of the filters in X-Gal buffer 37°C. Migration was quantitated by viewing under bright-field optics and counting the β-Gal stained cells in 10 fields (40X) from each of 3 filters for each experiment.

### Immunoprecipitations

Immunoprecipitation assays were done using 500 µg of whole-cell lysate as previously described (25). Rabbit polyclonal anti-R-Ras (Santa Cruz) and anti-FLNa (Santa Cruz) were used to imunoprecipitate the appropriate protein. Rabbit pre-immune serum was used as a control. Complexes were resolved by SDS-PAG and then analyzed by Western blot analysis.

### Western blot analysis

Extracts (30 µg) were resolved on 4–12% SDS-polyacrylamide gels and immunoblots done following standard procedures. The following antibodies were used: a rabbit polyclonal anti-FLNa antibody, a rabbit polyclonal anti-R-Ras antibody and an anti-human anti-Fibronectin antibody (Santa Cruz Biotechnology, Inc., Santa Cruz, CA).

### Immunostaining of Fibronectin Matrix

Clones expressing FLNa, FLNaΔ3, or vector control (FLNa-) were examined in matrix assembly assays as previously described [15], [28]. Briefly, 24 hours post plating human plasma FN (100 µg/well) was added and incubated for 24 h 37°C. Cultures were fixed in 3.7% paraformaldehyde 30 min RT, immunostained with a polyclonal rabbit anti-human FN antibody followed by goat anti-rabbit Rhodamine labeled IgG secondary (Sigma) and analyzed using a Leica TCS SP5 confocal microscope at 63X. Quantitation of fibronectin matrix was determined as previously described [15]. Briefly, transfected cells were cultured for 35 h with FN, washed, scraped into deoxycholate (DOC) buffer centrifuged at 20,000×g for 20 minutes 4°C, resuspended in SDS sample buffer, Western blotted for FN. Membranes were striped and reprobed with anti-vimentin antibody (Sigma clone Vim 13.2) as described [29].

### Immunofluorescence

24 h post-transfection, cells were incubated in serum free media for 24 h at 37°C and fixed in 4% paraformaldehyde in PBS for 30 min at RT. R-Ras was detected with a rabbit anti-R-Ras (C-19) antibody (Santa Cruz Biotechnology, Inc., Santa Cruz, CA) and an Alexa Fluor Goat Anti-Rabbit 594 secondary (Invitrogen, San Diego, CA). Confocal imaging was performed on a Leica TCS SP5 with 63X Oil Objective.

## Results

### R-Ras associates with FLNa

**Figure 1:**
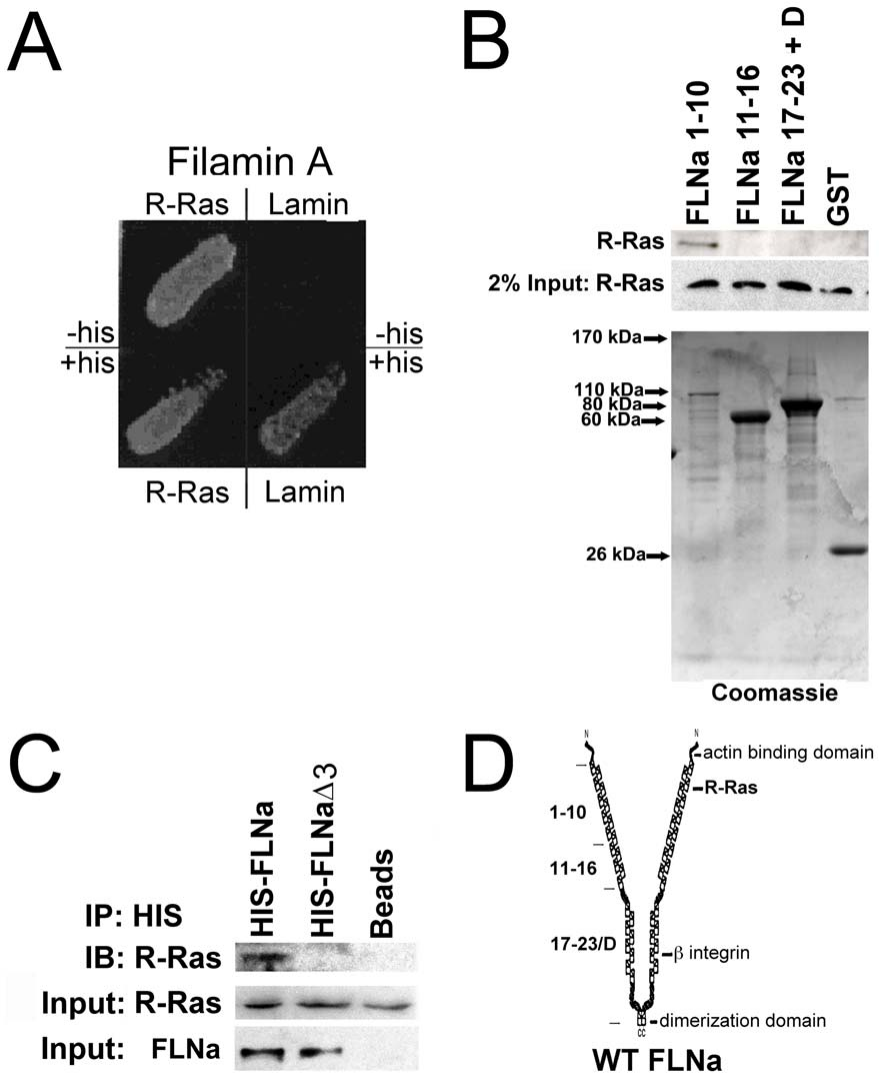
R-Ras associates with FLNa at repeat 3. **A**) Yeast two hybrid screen of R-Ras binding to FLNa. Colonies from + histidine (+his) plates obtained by co-transformation of the L40 yeast strain with BTM116R-Ras-CAAX^-^ or negative control BTM116-lamin C and VP16-FLNa (nucleotides 1699–1995). The plate was photographed after 3 days of growth. **B**) R-Ras binds FLNa. GST fusion proteins derived from indicated FLNa fragments were incubated with M2 (FLNa-) cell total lysates. Bound R-Ras was detected by Western blotting for R-Ras. Results are representative of 3 independent experiments. Coomassie stained gel containing purified FLNa fragments is shown to confirm FLNa fragment protein expression. **C**) R-Ras and FLNa co-immunoprecipitate in M2 melanoma cells. M2 cells were co-transfected with expression vectors for active R-Ras G38V, His-tagged wild type FLNa, His-tagged FLNaΔ3 (deletion of FLNa repeat 3) or vector alone. 48 h post transfection cells were lysed, immunoprecipitated (IP) with anti-His conjugated IgG beads or IgG beads alone and immunoprecipitates were analyzed by immunoblotting with an anti-R-Ras antibody. Input R-Ras and FLNa levels are shown (Bottom). Results are representative of 3 independent experiments. **D**) The domain structure of FLNa and the location of R-Ras binding site as determined by yeast two hybrid screen. The β-integrin subunits, the actin-binding domain, and the dimerization domain at the C-terminus have been established previously [5], [49], [50], [51]. FLNa fragments repeats 1–10, 11–16, and 17–23 + dimerization domain are shown on the left.

Expression of the small GTP-binding protein R-Ras enhances cell adhesion to extracellular matrix (ECM) substrates. This increase in adhesion results from enhanced integrin ligand-binding activity [16]. Because R-Ras is involved in intracelluar signaling to integrins, we set out to determine whether R-Ras associates with other cellular proteins. We used a yeast two-hybrid system utilizing R-Ras (with the CAAX sequence of R-Ras deleted) as bait. Lamin-C as bait was used as a negative control [30]. We used a mouse 9.5 embryonic cDNA library [31] as prey and screened 1×10^7^ colonies. Four clones from the R-Ras yeast two-hybrid screen encoded regions of filamin A (Fig. 1A). The R-Ras interacting filamin A (FLNa) clones had in common a segment between nucleotides 1699 and 1932, corresponding mainly to FLNa repeat 3 (nucleotides 1595–1882). FLNa did not bind to the negative control lamin C (Fig. 1A).

To further test the hypothesis that R-Ras associates with FLNa we used GST-filamin fusion proteins that correspond to 3 different regions of FLNa to pulldown R-Ras from a melanoma cell line (M2 cells). Indeed, R-Ras bound to the GST fusion protein corresponding to repeats 1–10, whereas it did not bind to the other regions tested (Fig. 1B). The region identified in the yeast two-hybrid screen is within repeat 3, which lies within the FLNa 1–10 fusion protein. Together these data support the hypothesis that R-Ras is in a complex with FLNa that requires FLN repeat 3.

We therefore made an expression construct of FLNa in which repeat 3 was deleted (FLNaΔ3). Cells were transfected with activated R-Ras in combination with FLNaΔ3 or wild type FLNa. FLNa and FLNaΔ3 were immunoprecipitated from cell lysates and examined for co-precipitation of R-Ras by immunoblotting. R-Ras was detected in FLNa immunoprecipitations confirming that R-Ras and FLNa are in a complex in mammalian melanoma M2 cells (Fig. 1C). Whereas FLNa lacking repeat 3 did not co-immunoprecipitate R-Ras (Fig. 1C), confirming that this is the site of association.

### R-Ras and FLNa co-localize in melanoma cells

**Figure 2:**
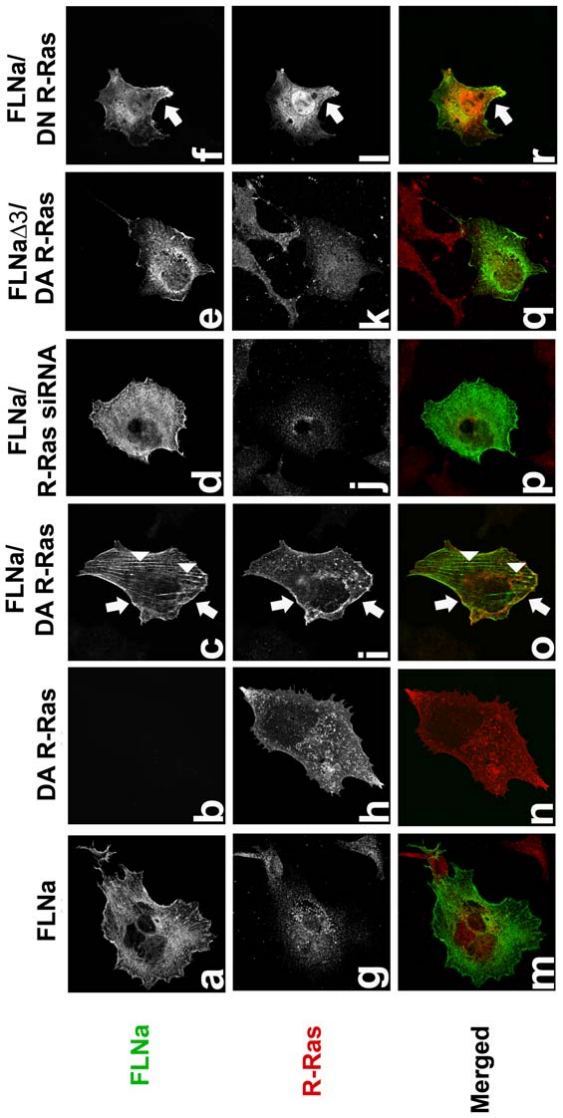
R-Ras and FLNa co-localize in M2 melanoma cells. FLNa and active R-Ras G38V partially co-localize in cells as examined by immunofluorescence confocal microscopy at 63X. Co-localization of FLNa and active R-Ras is displayed in yellow in the merged image. Arrows indicate co-localization. M2 (FLNa-) cells were transiently transfected with either wild type FLNa, dominant active R-Ras G38V (DA R-Ras), FLNa + DA R-Ras, FLNa + dominant negative R-Ras T43N (DN R-Ras), FLNaΔ3 (FLNa repeat 3 deleted) ± R-Ras, FLNa+R-Ras siRNA or FLNa + control scrambled siRNA and plated on FN coated glass slides. R-Ras was detected by immunostaining with an anti-R-Ras antibody followed by a Rhodamine conjugated secondary. FLNa and FLNaΔ3 were detected by immunostaining with an anti-FLNa antibody followed by Alexa Fluor 488 conjugated secondary. 63X. Data shown is representative of 3 independent experiments.

R-Ras associates with purified FLNa and they co-immunoprecipitate. To determine where this interaction occurs in cells we examined their sub-cellular localization by immunofluorescence. We transiently transfected M2 cells, which do not express FLNa, with active R-Ras G38V or dominant negative R-Ras T43N together with either FLNa or FLNaΔ3 (FLNa repeat 3 deleted). Active R-Ras G38V and FLNa co-localize in some regions of the cell and there is an apparent increase in these cells in the amount of FLNa at presumptive stress-fibers but not at the plasma membrane (Fig. 2c,i,o). In contrast, active R-Ras G38V and FLNaΔ3 did not co-localize (Fig. 2e,k,q) in agreement with our yeast two-hybrid and co-immunoprecipitation data. Dominant negative R-Ras (Fig. 2f,l,r) promotes more FLNa localization at the plasma membrane and FLNa does not go to the stress fiber-like striations.

We next examined endogenous R-Ras. Immunostaining for endogenous R-Ras demonstrated that R-Ras is found in the perinuclear region of cells (Fig. 2g), which is in agreement with recently published data that R-Ras is localized and active in endosomes [32]. Knockdown of endogenous R-Ras by siRNA results in FLNa at the plasma membrane and fewer FLNa striations (Fig. 2d,j,p).

### R-Ras binding to FLNa is required for melanoma cell migration

**Figure 3:**
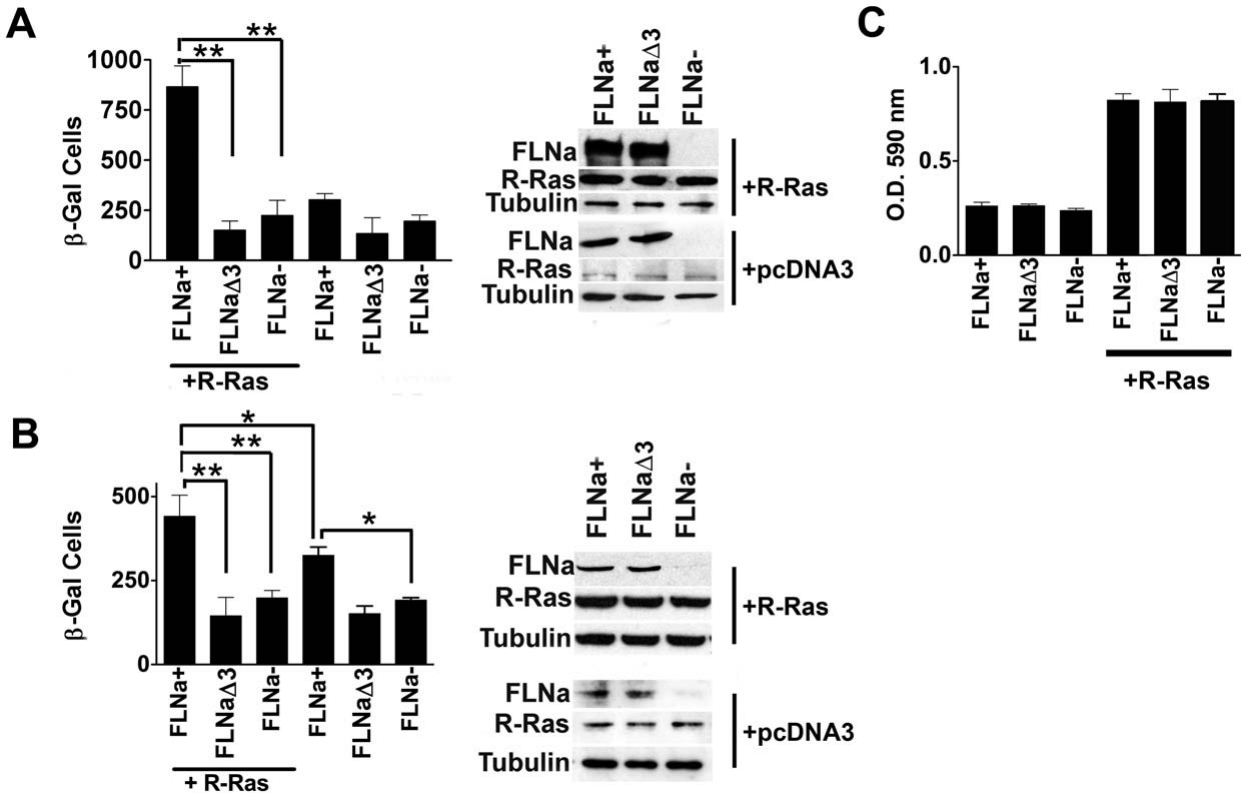
The association of R-Ras with FLNa enhances melanoma cell migration. **A**) Transwell migration assay was used to measure the migration of M2 melanoma cells in which dominant active R-Ras G38V was expressed in stable clones expressing wild type FLNa, FLNaΔ3, or vector control with no FLNa expression. The bottom side of filters were coated with 15 µg/ml FN. Migration was expressed as the number of migrated β-Gal expressing cells. Data represent the mean ± S.D. of three independent experiments. **Insert**: Western blot of stable M2 cell clones expressing wild type FLNa, FLNaΔ3 (deletion of FLNa repeat 3), or vector control with no FLNa expression and R-Ras G38V. Tubulin was used as a loading control. Data represent at least 3 independent experiments. Statistically significant differences are reported in the graph as *P* values (Student's *t*-test), **, *P*<0.01 **B**) Transwell migration assay was used to measure the migration of M2 (FLNa-) melanoma parental cell line in which FLNa, FLNaΔ3, or vector control and active R-Ras G38V and the transfection marker β-Gal were transiently transfected. Migration was expressed as the number of migrated β-Gal expressing cells. Data represent the mean ± S.D. of three independent experiments. Statistically significant differences are reported in the graph as *P* values (Student's *t*-test), **, *P*<0.01; *, *P*<0.04. **Insert**: Western blot of FLNa+, FLNaΔ3, or FLNa- with active R-Ras G38V expression in parental M2 (FLNa-) cell line or FLNa+, FLNaΔ3, or FLNa- cells with no active R-Ras G38V. Tubulin was used as a loading control. Data represent at least 3 independent experiments. **C**) Adhesion assay of cells expressing FLNa, FLNaΔ3, or no FLNa ± active R-Ras. Data represent at least 3 independent experiments. Top Insert: protein expression levels shown are for both A and C.

R-Ras enhances the migration and invasion potential of a number of cell types [18], [19], [23]. FLNa is also able to influence migration and M2 melanoma cells require FLNa for migration [7]. We therefore examined whether R-Ras binding to repeat 3 of FLNa influenced melanoma cell migration. M2 cells stably expressing empty vector (pcDNA3), wild type FLNa, or FLNaΔ3 (repeat 3 deleted) were produced (Fig. 3A insert). We assessed the effect of activated R-Ras on migration in these cells. Transwell, haptotactic migration assays were done in which the bottom side of the filters were coated with fibronectin (FN). Cells expressing both active R-Ras and FLNa migrated through the FN filters approximately 3-fold more than cells expressing R-Ras and FLNaΔ3 or active R-Ras alone (Fig. 3A). To confirm that the increase in cell migration in the stable cell lines was dependent upon the expression of both FLNa and R-Ras, we also repeated the experiment by transient transfection. Parental M2 cells were transiently transfected with active R-Ras G38V and either FLNa, FLNaΔ3 or vector alone in addition to β-gal. Transfected cells were then examined for migration through fibronectin coated transwell filters. Again, M2 cells expressing both R-Ras and FLNa migrated more than cells expressing R-Ras and FLNaΔ3 or R-Ras alone (Fig. 3B and insert). Moreover, co-expression of active R-Ras and FLNa significantly increased M2 migration 2-fold compared to FLNa alone (Fig. 3B and insert). These results suggest that FLNa mediates R-Ras signals to the cytoskeleton and/or to integrins to promote cell migration.

### R-Ras and FLNa cooperatively enhance α5β1 integrin activation in melanoma cells

**Figure 4:**
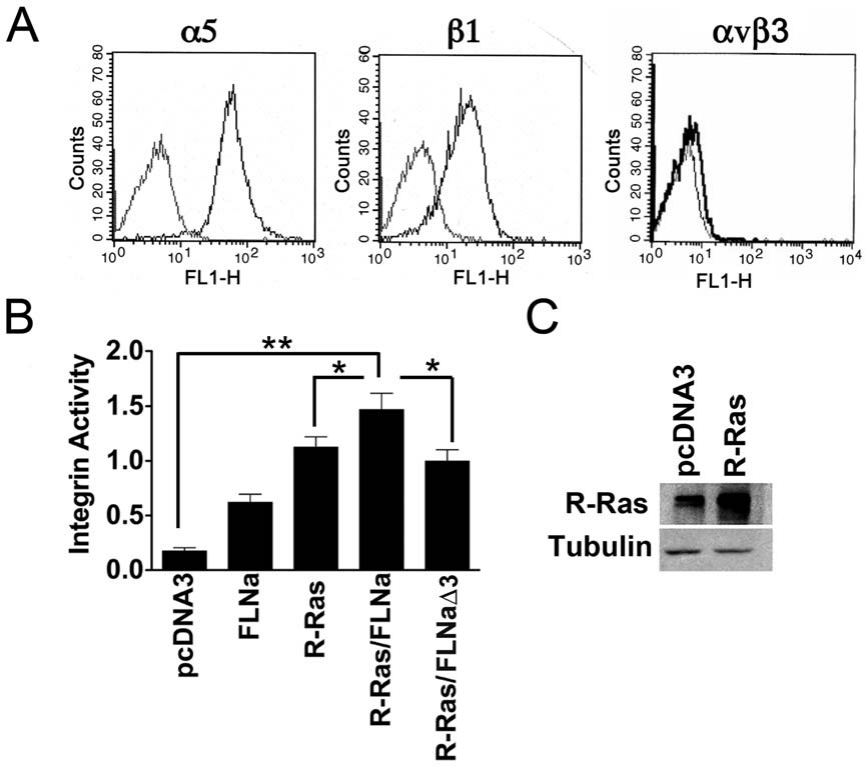
The association between R-Ras and FLNa enhances integrin activation. **A**) M2 cells express a subset of integrins on their surface. FACS analysis of a subset of integrins expressed on the surface of M2 cells. M2 cells express α5 and β1 subunits on their surface but not αvβ3. **B**) Forced expression of activated R-Ras promotes integrin activation in M2 cells as determined by the 3Fn-(9–11) activation assay. M2 (FLNa-) cells were transiently transfected with expression vectors encoding GFP as transfection reporter and FLNa, constitutively active R-Ras G38V alone or ±FLNa or FLNaΔ3. Cells were harvested and analyzed by two-color FACS for transfected cells and 3FN-(9–11) binding. Shown on the *Y-Axis* is mean activation index ±S.E. of three independent experiments. Constitutively expressed active R-Ras G38V and FLNa increased the activation index more than R-Ras alone. FLNaΔ3 did not block R-Ras-mediated activation compared with controls. Statistically significant differences are reported in the graph as *P* values (Student's *t*-test), *, *P*<0.05 **, *P*<0.04 **C**) Western blot of R-Ras levels when transiently transfected with R-Ras G38V. Tubulin was used as a loading control. Data represent at least 3 independent experiments.

FACs analysis showed that FLNa-deficient human melanoma M2 cells express α5β1 integrin, but not αvβ3 (Fig. 4A), both of which are high affinity (defined as activated) integrins [33], [34]. R-Ras is known to activate integrins and this can affect cell migration [16]. We therefore determined whether association of R-Ras with repeat 3 of FLNa could increase α5β1 integrin activation in M2 cells. Expression of constitutively active R-Ras G38V alone (Fig. 4B insert) induced α5β1 integrin activation in these cells as determined by the 3FN(F9-11) integrin activation assay [15], [35]. We found that in M2 cells expressing both active R-Ras and FLNa, integrin activity was significantly increased (Fig. 4B). Finally, co-expression of R-Ras and FLNaΔ3 (repeat 3 deleted) did not increase integrin activation in these cells (Fig. 4B). Thus R-Ras activation of integrins is enhanced by its interaction with FLNa at repeat 3. It is therefore possible that the enhancement of migration is due to changes in integrin activation. In agreement with published findings active R-Ras increased cell adhesion [16]; however we detected no difference in R-Ras mediated adhesion to FN in FLNaΔ3 or FLNa- expressing cells (Fig. 3C).

### R-Ras and FLNa cooperatively enhance fibronectin matrix assembly

**Figure 5:**
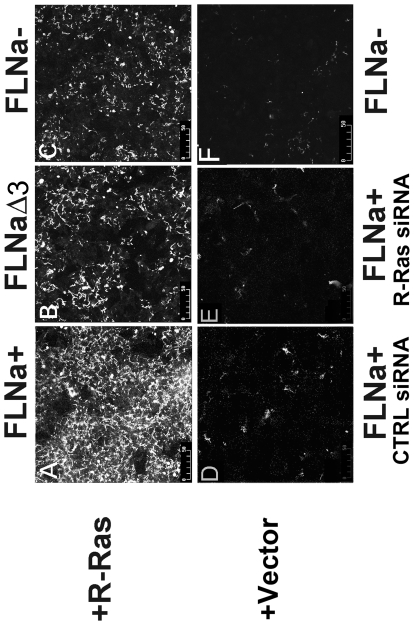
R-Ras binding to FLNa enhances fibronectin matrix assembly. Fibronectin matrix assembly in cultures expressing R-Ras G38V and either wild type FLNa (**A**), FLNaΔ3 (deletion of FLNa repeat 3; **B**), or cell cultures that do not express FLNa (FLNa-; **C**) were assessed. FLNa cultures expressing PcDNA3 vector and control scrambled siRNA (**D**) or knockdown of endogenous R-Ras using R-Ras siRNA (**E**) or control cultures not expressing FLNa (**F**) were also assessed. 24 hr after plating the culture media was replaced with media containing human FN (100 µg/ml) and cells were cultured for a further 48 hr before fibronectin matrix was assessed by immunostaining for FN. Each experiment was repeated a minimum of 3X.

**Figure 6:**
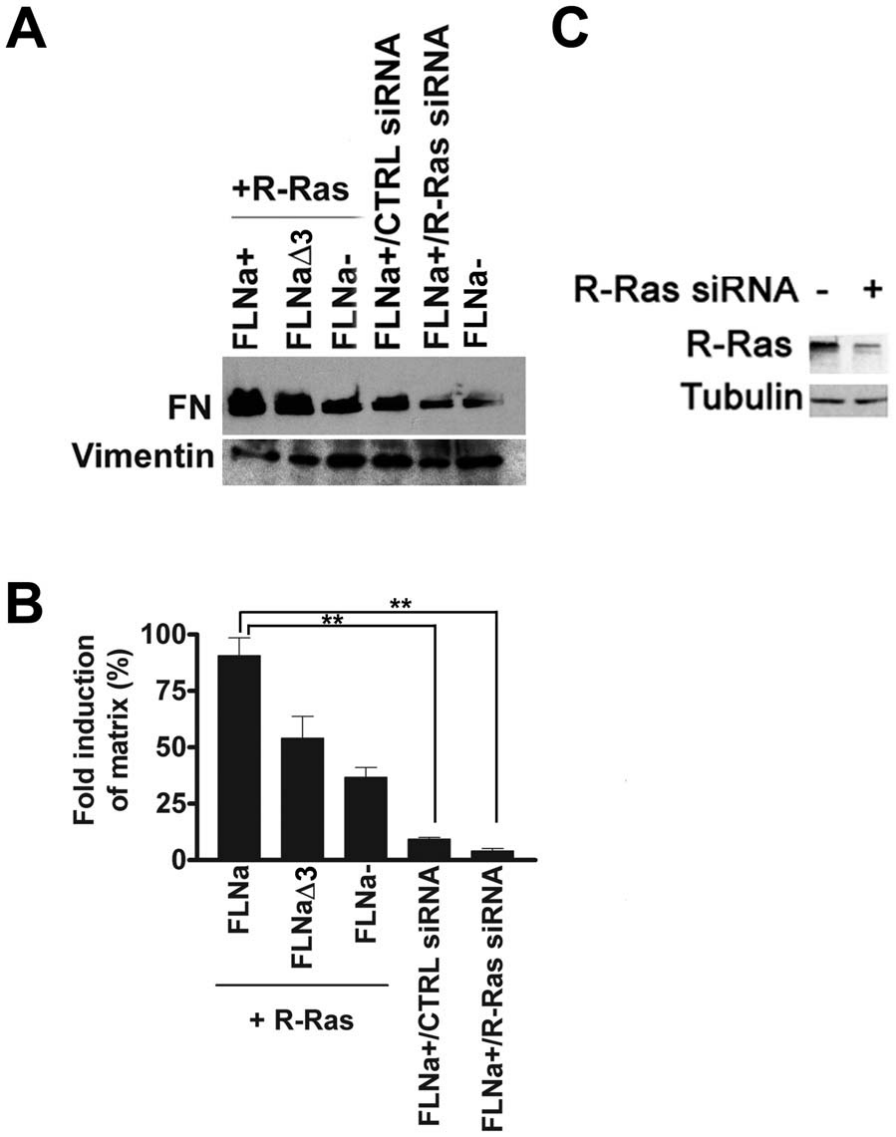
R-Ras binding to FLNa enhances deoxycholate-insoluble fibronectin matrix deposition. **A**) Deoxycholate (DOC)-insoluble fibronectin matrix formation was examined in M2 cultures expressing active R-Ras and either wild type FLNa, FLNaΔ3 (deletion of FLNa repeat 3), or M2 (FLNa-) control cell cultures. FLNa cultures with knockdown of endogenous R-Ras using R-Ras siRNA were assessed. Control scrambled siRNA and cultures not expressing FLNa (FLNa-) were also assessed. 48 hr after the addition of human FN to the wells, the DOC-insoluble material was subjected to SDS-PAGE analysis under reducing conditions and FN was detected by immunostaining. FN = Fibronectin. Vimentin was used as a loading control. **B**) Fold induction of matrix was normalized to M2 (FLNa-) control cultures. Graph shown represents pooled data from three independent experiments. Error bars represent S.E.M. Statistically significant differences are reported in the graph as *P* values (Student's *t*-test), **, *P*<0.05. **C**) Knockdown of endogenous R-Ras using siRNA or scrambled control siRNA is demonstrated via Western blot. 60 hr post transfection cells were lysed, run on SDS-PAGE gel and immunoblotted for R-Ras using a polyclonal anti-R-Ras antibody. Tubulin was used as a loading control. Results are representative of 3 independent experiments.

Activation of α5β1 integrin is required for fibronectin matrix assembly [33], [34], [36], [37], [38]. We therefore tested whether active R-Ras binding to FLNa repeat 3 would impact assembly of exogenously added fibronectin into a matrix in melanoma cells. Immunostaining for the presence of a fibronectin matrix (Fig. 5A–F) demonstrated that an increase in matrix formation occurred in the melanoma cultures expressing both activated R-Ras and FLNa (Fig. 5A) in comparison to cells expressing R-Ras and FLNaΔ3 (repeat 3 deleted; Fig. 5B) or R-Ras alone (Fig. 5C). Similarly, knockdown of endogenous R-Ras decreased fibronectin matrix assembly in FLNa expressing cells (Fig. 5E). M2 (FLNa-) cells did not develop a fibronectin matrix (Fig. 5F). In order to quantitatively measure the effects of R-Ras and FLNa co-expression on matrix assembly we isolated deoxycholate (DOC)-insoluble fibronectin matrix and immunoblotted the amount of FN matrix on the various cell types. This similarly revealed that M2 cells expressing both active R-Ras and FLNa contained an increase of DOC-insoluble fibronectin compared to cultures expressing R-Ras and FLNaΔ3 (repeat 3 deleted) or R-Ras alone (Fig. 6). Again, knockdown of endogenous R-Ras levels via siRNA (Fig. 6) reduced DOC-insoluble matrix formation in FLNa expressing cells (Fig. 6). These results indicate that FLNa enhances the ability of R-Ras to promote fibronectin matrix assembly in melanoma cells.

## Discussion

R-Ras regulates multiple cellular functions including adhesion and migration. We used a yeast two-hybrid screening approach to identify FLNa as a R-Ras binding protein. We report that R-Ras associates with FLNa in a manner dependent on FLNa repeat 3. Moreover, these proteins partially co-localize in melanoma cells. We further show that the complex is functional in that R-Ras and FLNa expressing melanoma cells demonstrate significant increased migration. Deletion of FLNa repeat 3 abrogates this function. Moreover, integrin activation and fibronectin matrix assembly increased in melanoma cells expressing active R-Ras and full length FLNa. These results suggest that FLNa may relay R-Ras signals to integrins to regulate migration and matrix assembly.

R-Ras interacts with a number of effector proteins including Raf1, NORE1, RLIP76, RalGDS, ORP3 and PI3-kinase [39]. R-Ras alters cell motility through Rac and Rho (22) and the R-Ras effector RLIP76 is required for R-Ras mediated enhancement of Rac-dependent cell migration (23). Based upon our data, R-Ras mediated migration in melanoma cells requires R-Ras binding directly to FLNa repeat 3. How this relates to the requirement for Rac and RLIP76 remains unclear. It may be that FLNa forms a scaffold to support or localize these proteins during migration.

A primary mechanism for driving tumor metastasis has been proposed to be through enhanced endocytic trafficking of integrins [40]. R-Ras activates RalA on endosomes (34) and R-Ras has also been localized to endosomes in an EGF-dependent manner (31) suggesting that R-Ras is active in the perinuclear region of cells. Our data indicate that R-Ras and FLNa co-localize at the plasma membrane. FLNa is involved in regulating caveolae internalization and trafficking [41]. Therefore R-Ras may be poised to enhance FLNa function in endosome formation and thereby enhance integrin-mediated migration. In addition, FLNa binding to the β integrin tails blocks cell migration (4) and integrin activation [42], [43]. Thus, R-Ras may recruit FLNa away from the β integrin tails and thereby promote cell migration.

Fam38A mediates integrin activation by recruiting R-Ras to the ER, which activates the calcium-activated protease calpain [44]. Recently, FLNa was shown to be involved in integrin β1 activation via its interaction with vimentin and protein kinase C-epsilon [42]. We observed an increase in integrin activation and fibronectin matrix assembly upon co-expression of active R-Ras and FLNa suggesting that this interaction may be important for integrin-mediated migration and integrin activation. Because the known R-Ras effectors are not critical for regulating integrin activation [45] it may be that FLNa supports R-Ras's ability to increase integrin activation. Moreover, FLNa is important in the initiation step of migration and deletion of the FLNa binding site for integrins (FLNa repeats 19–21) has no effect on migration [46]. This suggests that migration is not dependent upon FLNa binding to integrins but rather some other region of the protein. We found that R-Ras association with FLNa through repeat 3 is required for migration. Thus, FLNa-dependent migration requires R-Ras interaction with repeat 3 but is independent of FLNa-integrin binding. Furthermore, in our transient transfection experiments, where the expression levels of FLNa were similar, we observed a significant increase in melanoma cell migration upon co-expression of active R-Ras suggesting that R-Ras and FLNa work in tandem to support migration. R-Ras expression promoted increased cell adhesion to FN; however we did not observe any changes in adhesion upon FLNa expression. This may be due to the fact that we examined FN-mediated adhesion and not collagen-mediated adhesion as FLNa is reported to play a role in cell adhesion and spreading on collagen [42], [47]. Whether the functional association of R-Ras with FLNa is required for adhesion and spreading to collagens remains to be determined.

Tumor cell metastasis from a primary tumor into other tissues is the cause of most cancer deaths [3]. Changes in cell-extracellular matrix (ECM) and cell-cell interactions are associated with the progression of primary melanomas to malignancy. The Ras GTPase superfamily regulates cell proliferation, adhesion, migration, and vesicle transport and are often mutated in human cancers [48]. Our findings that concomitant expression of active oncogenic R-Ras and full-length wild type FLNa induced melanoma cell migration suggest that FLNa may relay R-Ras signals to integrins to regulate migration and subsequently contribute to tumor metastasis.
